# 

**Published:** 1894-10

**Authors:** 


VOL. XV.] PUBLISHED MONTHLY AT THREE DOLLARS A YEAR. SINGLE COPIES, THIRTY CENTS. [No. 5.

Copyright, 1893, by W. A. Conklin and R. S. Huidekoper.
Printed for the Editors by the E. SCOTT Co.

### THE JOURNAL

OF

### COMPARATIVE MEDICINE

AND

### VETERINARY ARCHIVES.

PHILADELPHIA.

EDITED AND PUBLISHED BY

RUSH SHIPPEN HUIDEKOPER, M. D., Veterinarian, (Alfort),

W. A. CONKLIN, Ph.D., D. V. S.

W. HORACE HOSKINS, D. V. S. A. W. CLEMENT, D. V. S.

OCTOBER, 1894.

All communications and payments should be addressed to

**Dr. W. HORACE HOSKINS, 3452 Ludlow Street, Philadelphia, Pa.**

**Copies may be had in London of BAILLIERE, TINDALL & COX.**

